# Biomechanical comparative study of midline cortical vs. traditional pedicle screw trajectory in osteoporotic bone

**DOI:** 10.1186/s12891-023-06502-x

**Published:** 2023-05-18

**Authors:** Stefan Schleifenbaum, Ann-Cathrin Vogl, Robin Heilmann, Nicolas Heinz von der Hoeh, Christoph-Eckhard Heyde, Jan-Sven Jarvers

**Affiliations:** 1 Department of Orthopedic, Trauma and Plastic Surgery, University of Leipzig, Liebigstraße 20, D-04103 Leipzig, Germany; 2 ZESBO – Zentrum zur Erforschung der Stuetz- und Bewegungsorgane, Semmelweisstraße 14, D-04103 Leipzig, Germany

**Keywords:** Cortical bone trajectory (CBT), Biomechanical analysis, MC, Osteoporosis, Patient-specific placement guide

## Abstract

**Introduction:**

In lumbar spinal stabilization pedicle screws are used as standard. However, especially in osteoporosis, screw anchorage is a problem. Cortical bone trajectory (CBT) is an alternative technique designed to increase stability without the use of cement. In this regard, comparative studies showed biomechanical superiority of the MC (midline cortical bone trajectory) technique with longer cortical progression over the CBT technique. The aim of this biomechanical study was to comparatively investigate the MC technique against the not cemented pedicle screws (TT) in terms of their pullout forces and anchorage properties during sagittal cyclic loading according to the ASTM F1717 test.

**Methods:**

Five cadavers (L1 to L5), whose mean age was 83.3 ± 9.9 years and mean T Score of -3.92 ± 0.38, were dissected and the vertebral bodies embedded in polyurethane casting resin. Then, one screw was randomly inserted into each vertebra using a template according to the MC technique and a second one was inserted by freehand technique with traditional trajectory (TT). The screws were quasi-static extracted from vertebrae L1 and L3, while for L2, L4 and L5 they were first tested dynamically according to ASTM standard F1717 (10,000 cycles at 1 Hz between 10 and 110 N) and then quasi-static extracted. In order to determine possible screw loosening, there movements were recorded during the dynamic tests using an optical measurement system.

**Results:**

The pull-out tests show a higher pull-out strength for the MC technique of 555.4 ± 237.0 N compared to the TT technique 448.8 ± 303.2 N. During the dynamic tests (L2, L4, L5), 8 out of the 15 TT screws became loose before completing 10,000 cycles. In contrast, all 15 MC screws did not exceed the termination criterion and were thus able to complete the full test procedure. For the runners, the optical measurement showed greater relative movement of the TT variant compared to the MC variant. The pull-out tests also revealed that the MC variant had a higher pull-out strength, measuring at766.7 ± 385.4 N, while the TT variant measured 637.4 ± 435.6 N.

**Conclusion:**

The highest pullout forces were achieved by the MC technique. The main difference between the techniques was observed in the dynamic measurements, where the MC technique exhibited superior primary stability compared to the conventional technique in terms of primary stability. Overall, the MC technique in combination with template-guided insertion represents the best alternative for anchoring screws in osteoporotic bone without cement.

## Background

Pedicle screw instrumentation is the most common surgical technique in lumbar spine stabilization. Cement augmentation is commonly used in osteoporotic spine [10, 16, 41]. Nevertheless, several possible complications like pulmonary embolism, cardiovascular complication or cement leakage can occur and moreover revision surgery can be problematic [24, 34, 41]. To improve pedicle screw fixation in bone of compromised quality, different screw designs and insertion techniques regarding screw trajectory modifications were developed [16, 35]. Santoni et al. [34] introduced in 2009 the cortical bone trajectory (CBT) fixation approach. The entry of this trajectory starts at the pars interarticularis medially and follows a craniolaterally direct path through the pedicle. The medially directed traditional trajectory (TT), on the other hand, has a lateral starting point and uses a transpedicular path through the anatomic axis of the pedicle [31]. Correspondingly, TT pedicle screws achieve their stability apart from the pedicle in cancellous bone, often resulting in a loss of stability in osteoporotic patients if no bone cement is used. CBT screws, in contrast, are characterized by increased screw thread contact with cortical bone [27]. A further option is the midline cortical (MC) approach, which is derived from the CBT technique. The entry points are sufficiently distant from the adjacent facet joints and the trajectory follows from the pars interarticularis to the inferior edge of the pedicle. Hence, the MC approach realizes the use of longer screws with a minimum length of 40 mm while the original CBT method grants a screw length usually no longer than 25–30 mm [28]. In a recent biomechanical study a biomechanical superiority of MC compared to CBT could be shown. Furthermore, it has been demonstrated, that MC may serve as a viable alternative to cement augmented screws [14]. As reaching the correct MC trajectory can be challenging, the use of a patient specific screw placement guide has proved its value [11, 14, 23].

The objective of this biomechanical study was to conduct a comparative investigation between the MC technique, utilizing patient specific placement guides and non-cemented pedicle screws implanted via freehand technique (TT) with regards of their pullout forces and anchorage properties under sagittal cyclic loading according to the ASTM F1717 test protocol.

## Methods

### Specimen and grouping

Like in our recent study [14] five human cadaveric specimens, especially L1 to L5, without destructive pathologies (fractures, tumor) were obtained in fresh and anatomically unfixed condition. All donors originated from the Institute of Anatomy of the University of Leipzig and had given written informed consent to dedicate their bodies to medical education and research purposes. Being part of the body donor program regulated by the Saxonian Death and Funeral Act of 1994 (3rd section, paragraph 18, item 8), institutional approval for the use of the post-mortem tissues of human body donors was obtained. The authors declare that all experiments were performed according to the ethical principles of the Declaration of Helsinki.

During dissection, all vertebrae were separated into single levels. Muscular and soft tissue was removed from each vertebra while preserving its anatomy. The specimens were stored at − 83 °C until testing. A dual-energy X-ray absorptiometry (DXA) analysis was performed to determine the bone mineral density (Hologic Delphi A QDR Series, Bedford, USA). In addition, a low-dose computed tomography (CT) scan (PHILIPS Brilliance iCT 256, Philips Healthcare, Cleveland, OH, USA) of all specimens was taken for the exclusion of bone defects and for preoperative planning.

### Preoperative planning and preparation

The diameter and the lengths of TT screw was identified by an experienced surgeon using the CT scans. Planning for the screws used in the MC technique followed the same protocol as Jarvers et al. [14]. An individual drilling template was used for each screw using the MySpine® technique (Medacta International SA, Castel San Pietro, Switzerland) The test procedure and implantation were similar to that of Jarvers et al. [14] to ensure comparability between the studies. The thawed lumbar vertebrae were embedded in an aluminium cylinder using RenCast® FC52/53 Isocyanate mixed in a ratio of 1:1:3 with RenCast® FC52t Polyol and Filler DT 082 (Huntsman Corporation, Salt Lake City, UT, USA) and were instrumented by the same experienced surgeon. The TT Screws were implanted freehand, whereas the patient-specific placement guide (MySpine®, Medacta International SA, Castel San Pietro, Switzerland [Cecchinato, Farshad,Lamartina]), was used to guide the drilling of the MC trajectory. For both techniques Medacta Universal Screw Technology (M.U.S.T., Medacta International SA, Castel San Pietro, Switzerland) was used for all pedicle screws.

### Biomechanical testing

All specimens were divided into two groups for static and dynamic examination, respectively.

**Fig. 1:**
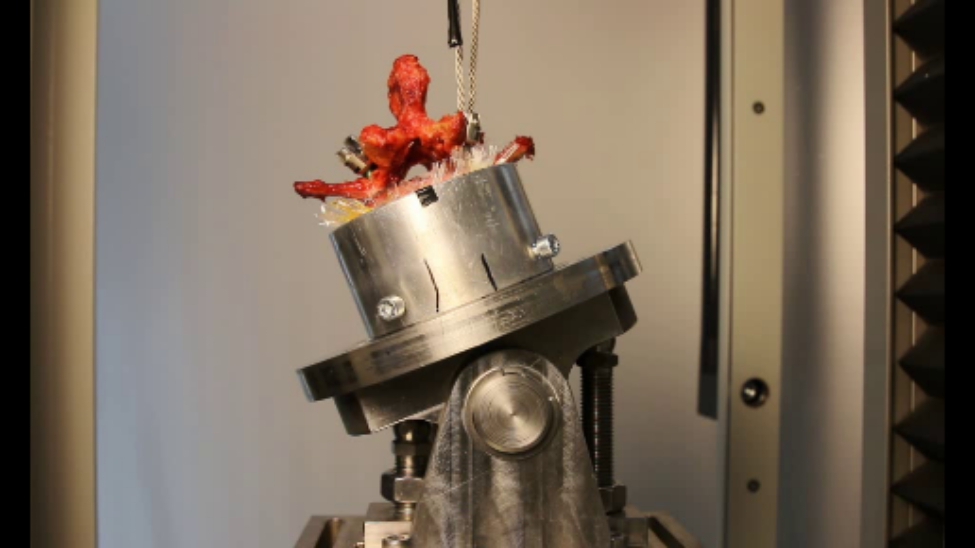
Experimental pull-out setup

**Fig. 2:**
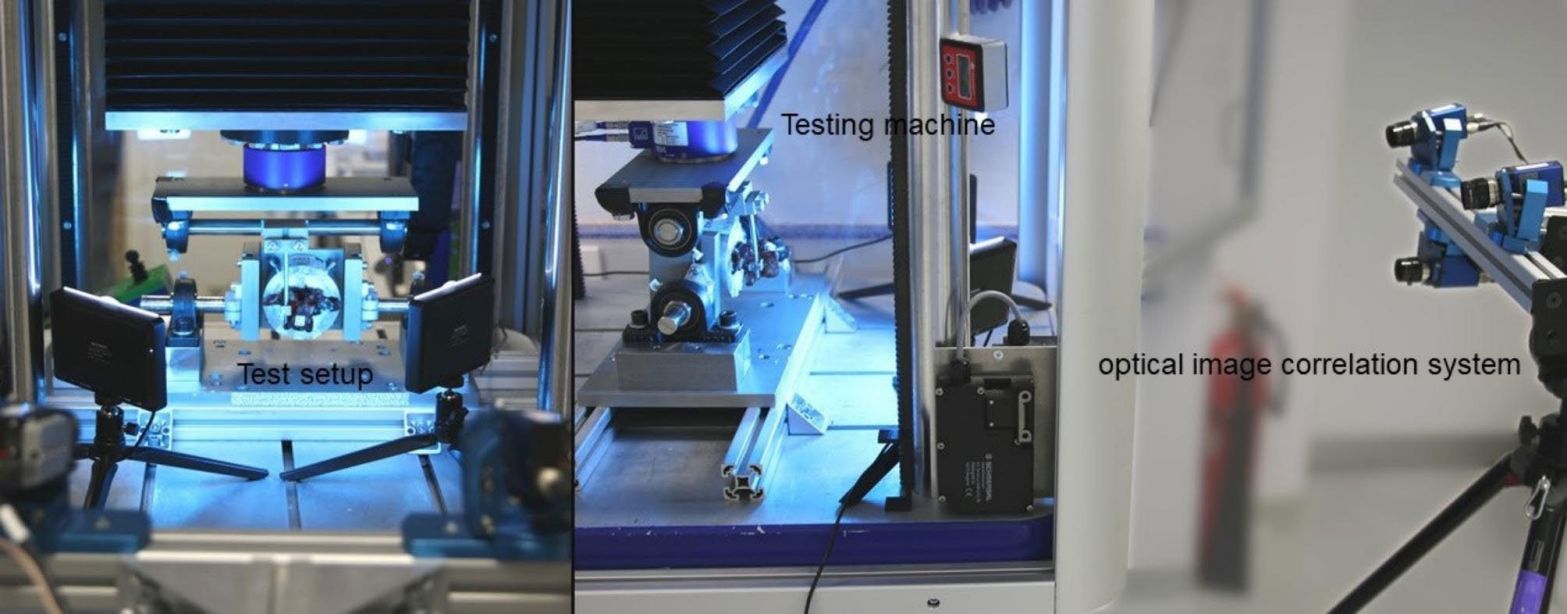
Experimental setup of the dynamic test with a vertebral body and the optical measuring system

Thereupon the quasistatic tests (group 1) were performed with L1 and L3 and the dynamic tests (group 2) with L2, L4 and L5. Concerning the static testing [14], the screws were pulled out axially through a customized designed experimental setup (Figs. 1 and 2) which guarantees a shear force-free pullout with mounting on x and y bearing and an axial alignment by means of a rocker. For the tests we were using a uniaxial testing machine (Type Z020, ZwickRoell GmbH & Co. KG Ulm, Germany, load cell Xforce HP 2,5 kN, Accuracy class 0,2) with a testing speed of 5 mm/min. The extraction was always carried out randomly so that either the TT or MC screw was pulled out first.

For the dynamic tests using a servo-pneumatic uniaxial testing machine (Type 2082/000, DYNA-MESS Prüfmaschinen GmbH Aachen, Germany with a load cell HBM MCS10-010-6 C 10 kN, Accuracy class 0,2), a self-developed test set-up was used and the same procedure as in Jarvers et al. was chosen [14]. The cyclic test was conducted using the same parameters of 10,000 cycles between 10 and 110 N at 1 Hz. The relative screw movement between vertebral and screw head was recorded for specific cycles by using optical image correlation system (Q400, LIMESS Messtechnik und Software GmbH, Krefeld, Germany, Measurement Accuracy/Resolution 0.01 pixel for 3D motions). Following the dynamic examinations, the screws were pulled out to determine the remaining pull-out force after cyclic loading.

### Statistical analyses

The data were compared descriptively using Excel 2016 (Microsoft Corporation, Redmond, WA, USA). Furthermore, the data were examined statistically with the Wilcoxon test and the use of SPSS 24.0 (IBM, Armonk, NY, USA). The level of statistical significance was set at p < 0.05.

## Results

The mean age of the body donors was 78.55 ± 11.74 years (4 males and 1 woman) with a mean BMD of 0,65 ± 0,04 g/cm² and T Score − 3.92 ± 0.38.

### Static

**Fig. 3:**
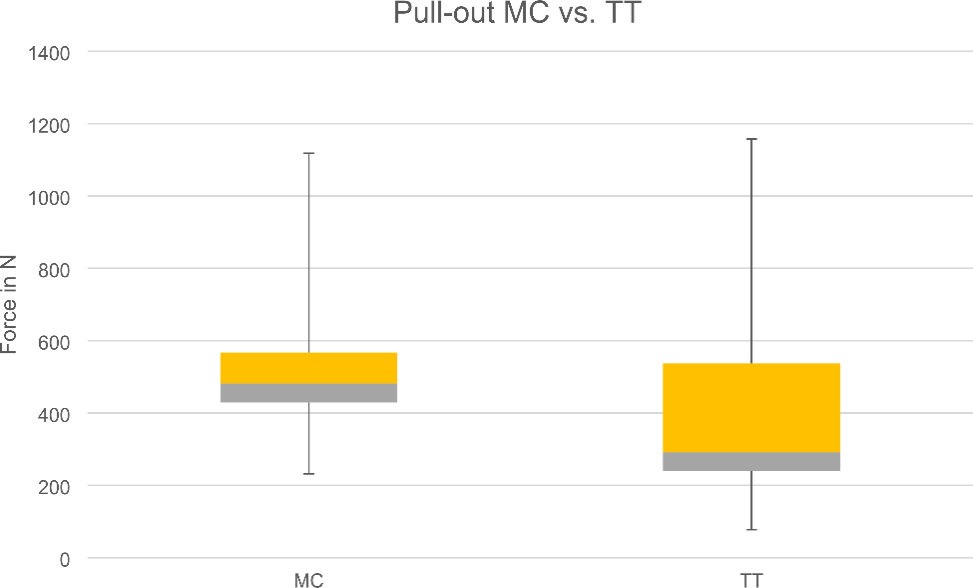
Boxplot of screw pull-out forces (MC vs. TT screw)

In the static pull-out tests, all 20 vertebrae were successfully tested with both screws. The L1 and L3 vertebrae showed an average higher pull-out force with the MC screws (535.45 ± 249.85 N) than the TT screws (448.83 ± 319.66 N) (Fig. 3). In a direct pairwise comparison (p = 0.646) of both techniques for each vertebra, five out of ten times the MC technique achieved higher pull-out forces than the TT technique, which were superior five times.

### Dynamic

**Fig. 4:**
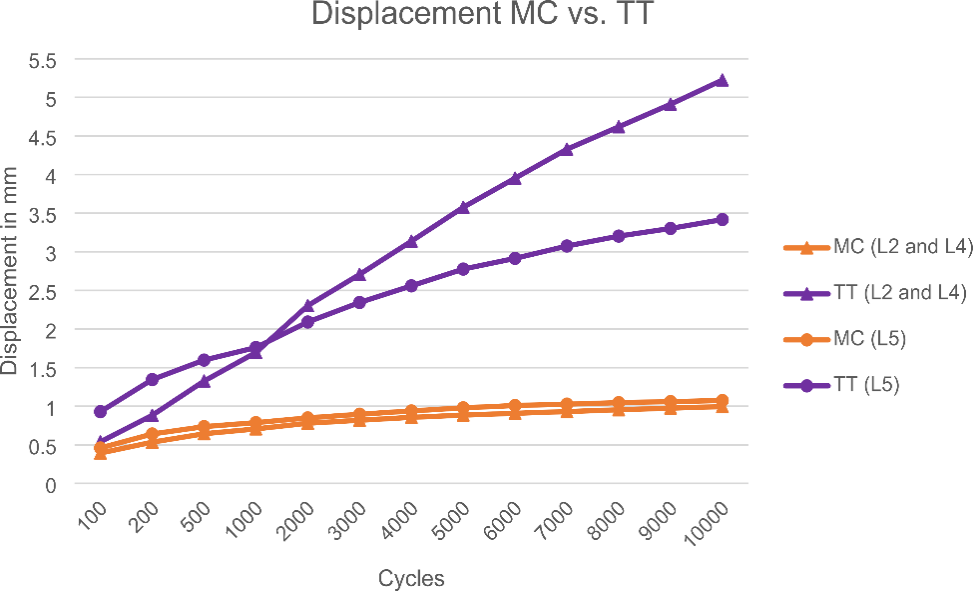
Displacement of screw head relative to its vertebra (MC vs. TT screw)

**Fig. 5:**
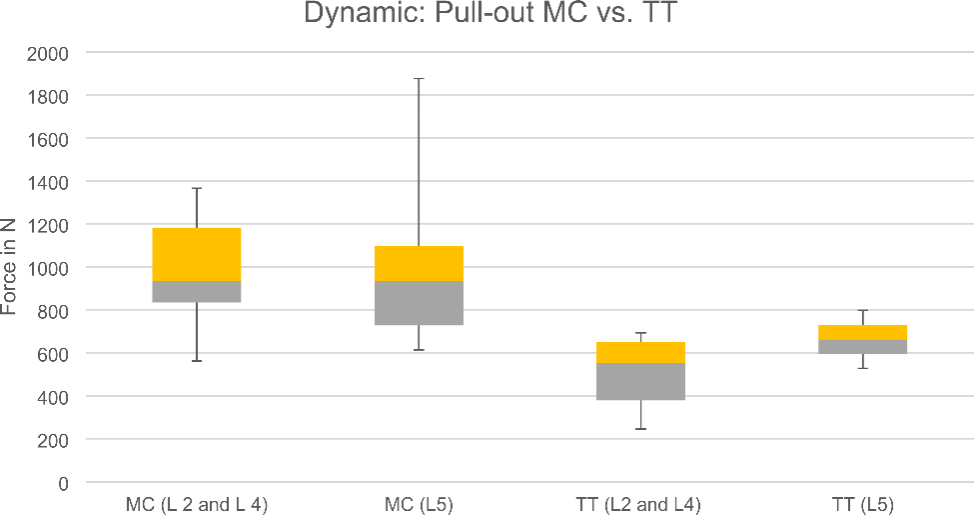
Boxplot of screw pull-out forces after dynamic testing of L2, L4 and L5 (MC vs. TT screw)

**Fig. 6:**
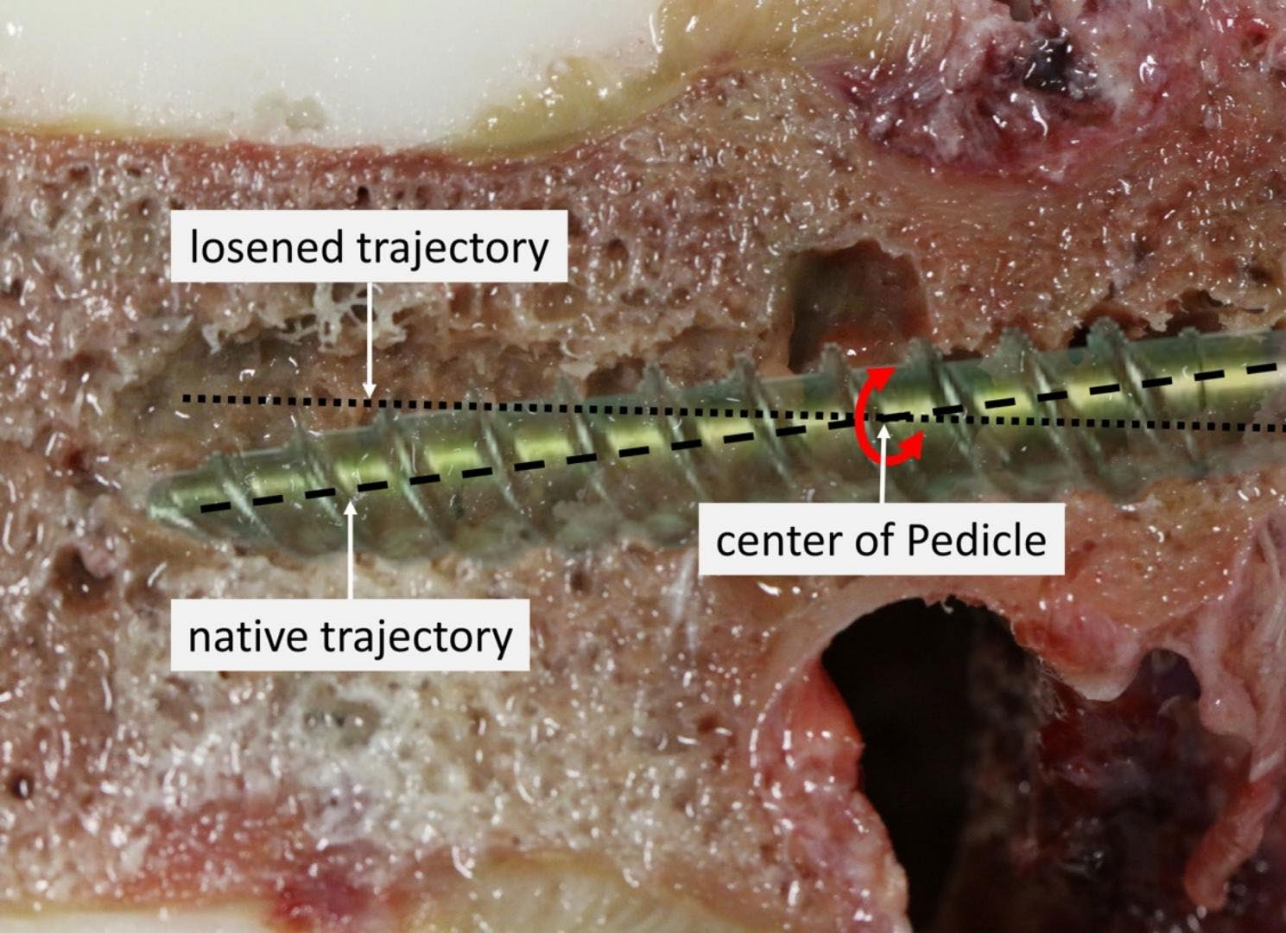
Wiper movement of the TT screw after dynamic testing

In the dynamic tests, no signs of failure or loosening were observed in the MC screws. In the TT technique, seven screws loosened before reaching 10,000 cycles, and loosening occurred between 99 and 6,300 cycles. The mean range of motion was only analysed of those screws that reached total 10,000 cycles. The optical measurement resulted in a lower range of motion of the MC screws compared to the TT screws (Fig. 4). The subsequent pull-out test was carried out only on those screws of the L2, L4 and L5 vertebrae that had previously also successfully completed the 10,000 cycles (23/30). In comparison, the pull-out tests show an overall higher pull-out strength of the MC version with 1002.58 ± 355.65 N compared to the TT version with 546.94 ± 186.42 N. In the paired comparison of the vertebrae where the TT screws completed 10,000 cycles, the MC screws exhibited consistently higher pull-out strength (p = 0.028). For comparability with the study by Jarvers et al. [14] L5 was compared separately with comparable results, with a failure during cyclic loading from 3 TT screws of 5. The range of motion of the TT screws was larger than that of the MC screws. The MC screws also achieved higher pull-out forces after dynamic testing (p = 0.180) (Fig. 5). Figure 6 depicts a cut-away vertebra of the failed TT screw and the loosening area.

## Discussion

Spinal stabilizations in osteoporotic patients remain challenging with various instrumentation techniques available. According to the literature [4, 5, 13, 36, 37, 39], pedicle screw augmentation of the posterior lumbosacral spine has been considered as the gold standard. This was underlined by several biomechanical studies, demonstrating an increased pull-out strength [4, 5, 13, 39] and good clinical outcomes with low revision rates in clinical mid- and long-term studies [2, 7, 8]. However, there are several possible complications, such as cement leakage, pulmonary embolism or cardiovascular complication. Additionally, exothermic properties or complications during screw removal have been reported [13, 24, 34, 41].

Therefore, alternatives were evaluated. Santoni et al. [34] first described the advantages of the CBT as a less invasive technique. In contrast to the TT these shorter and thinner screws are characterized by their extensive contact with the solid cortical bone leading to an increased fixation strength. In their study, CBT screws demonstrated a 30% greater uniaxial pull-out strength and an equivalent strength against toggle loading as compared to non-augmented TT screws [34]. The fixation strength simulating more physiological conditions using cyclical loading and subsequent orthogonal screw pull-out was investigated by Baluch et al. [3]. They also demonstrated the superior resistance of CBT screws. Matsukawa et al. [30] evaluated the insertional torque in vivo using the CBT and TT fixation approach, respectively. Their comparative study of both techniques showed a significant difference in the mean maximum insertional torque for the benefit of the CBT screws. In a finite element analysis of Matsukawa et al. [27] the results showed a mean 27.8% higher resistance to cephalocaudal loading, 26.4% greater mean pull-out strength and 140.2% stronger stiffness to mediolateral loading than non-augmented TT screws. While Wray et al. [40] reported comparable mechanical fixation properties of both approaches in their cadaveric biomechanical study including pull-out and toggling testing, contrary results were achieved by Akpolat et al. [40]. In their study non-augmented TT screws had a better fatigue performance than CBT screws in osteoporotic vertebrae.

The optimal size and length of the screws for use in CBT is a commonly debated topic. Matsukawa et al. [31] analyzed the ideal screw size for optimal fixation to significantly enhance screw’s fixation strength. They suggested the use of longer cortical screws to decease the mechanical stress and to improve vertebral load transmission. Their finite element study demonstrated biomechanical superiority with a long trajectory and maximum cortical purchase. Thus, the MC or “long CBT” screw, which is directed towards a more anterior position of the vertebral body compared to the original CBT, is recommended. Regarding to their results the ideal CBT screw should have a diameter larger than 5.5 mm and a length longer than 35 mm (standard size) [31]. These results were supported by the recent study of Jarvers et al. [14], where it was shown that MC screws showed better fatigue and pull-out forces than CBT. The authors concluded that compared to CBT screws and when cement reinforcement should be avoided, MC is a promising alternative in osteoporotic bone [14].

Recent studies have underlined that longer and deeper placement of CBT screws not only provides biomechanical advantages but also promotes bone fusion [1, 17, 18, 22, 29]. Through a less extensive dissection the CBT (or MC) screws include the ability to preserve more of the patient’s soft tissue in comparison to traditional pedicle screws. This result in potentially less intraoperative blood loss, reduced operative time, less postoperative pain, and reduced risk of intraoperative complications regarding an affection of the spinal canal with a lateral to medial trajectory [9, 40].

Moreover, these advantages can be relevant in obese patients, as extensive paraspinal dissection can be limited or more cortical bone purchase is desirable such as in osteoporotic patients. Recently both static and dynamic biomechanical studies have validated the superior pullout strength of CBT and MC versus the traditional pedicle screws [9, 25, 27, 28, 33, 40].

In this biomechanical study, the pullout tests demonstrated that the MC screws have a higher pullout force compared to TT screws. Direct pairwise comparison of the two techniques for each vertebra shows advantage for MC technique. The two techniques show a higher pull-out force in 5 cases each, so that no direct advantage can be derived. The main difference was observed in the more practical dynamic tests, where only the TT technology loosened before reaching 10,000 cycles. Out of the 15 screws, 7 loosened between 99 and 6300 cycles. In addition, the successfully tested screws showed a lower range of motion of the MC screws compared to the TT screws, as well as a higher pull-out force of the MC screws. Additionally, dynamic testing provides a better indication of screw stability as it better reflects reality. This point can be illustrated by showing the wiper movement of the screw from the test.

In comparison to the TT placement, achieving the correct trajectory in CBT or MC can be challenging for surgeons. The screw path has to be reached in the denser bone with fewer anatomical landmarks available. Intraoperative fluoroscopic support is needed to enhance accuracy and safety apart from high-level surgical skills. Optical navigation and robot-assisted systems are the most alternative advanced technologies achieving high accuracy of screw placement linked with minimal invasiveness. However, the additional time, the radiation exposure and the high cost for installation and maintenance, required space and personnel for operation, must be considered [12, 26, 32]. In this context the use of a patient specific screw placement guide with a preplanned screw trajectory has been considered as a promising approach [6, 15, 19–21, 31, 35, 38].

However, there are some limitations to this study that need to be considered. Firstly, the study sample size is very limited and the varying sample sizes of the individual groups should be critically reviewed. Therefore, a more extensive evaluation using equal sample sizes is desirable to confirm these results. Moreover, the position of the embedded vertebrae is not physiological, but a standardized procedure regarding the literature. Finally, for biomechanical testing, only cadaveric specimens were used, and the results may not necessarily translate to in vivo settings.

### Conclusion

The highest pullout forces were achieved by the MC technique. The main difference between the techniques was shown in the dynamic measurements where the MC technique was clearly superior to the conventional technique in terms of primary stability. Overall, the MC technique in conjunction with insertion using a template seems to represent the best alternative for anchoring screws in osteoporotic bone without cement.

## Data Availability

The datasets used and/or analysed during the current study are available from the corresponding author on reasonable request.

## Abbreviations

BMD: Bone mineral density
CBT: Cortical bone trajectory
CT: Computed tomography
DXA: Dual-energy X-ray absorptiometry
L: Lumbar vertebra
MC: Midline cortical
PMMA: Polymethylmethacrylate
SD: Standard deviation
TT: Traditional trajectory
